# Factors and Models Associated with the amount of Hospital Care Services as Demanded by Hospitalized Patients: A Systematic Review

**DOI:** 10.1371/journal.pone.0098102

**Published:** 2014-05-30

**Authors:** Catharina J. van Oostveen, Dirk T. Ubbink, Judith G. Huis in het Veld, Piet J. Bakker, Hester Vermeulen

**Affiliations:** 1 Department of Surgery, Academic Medical Center, Amsterdam, The Netherlands; 2 Department of Quality Assurance & Process Innovation Academic Medical Center, Amsterdam, The Netherlands; 3 Amsterdam School of Health Professions, University of Amsterdam, Amsterdam, The Netherlands; University of Maryland, School of Medicine, United States of America

## Abstract

**Background:**

Hospitals are constantly being challenged to provide high-quality care despite ageing populations, diminishing resources, and budgetary restraints. While the costs of care depend on the patients' needs, it is not clear which patient characteristics are associated with the demand for care and inherent costs. The aim of this study was to ascertain which patient-related characteristics or models can predict the need for medical and nursing care in general hospital settings.

**Methods:**

We systematically searched MEDLINE, Embase, Business Source Premier and CINAHL. Pre-defined eligibility criteria were used to detect studies that explored patient characteristics and health status parameters associated to the use of hospital care services for hospitalized patients. Two reviewers independently assessed study relevance, quality with the STROBE instrument, and performed data analysis.

**Results:**

From 2,168 potentially relevant articles, 17 met our eligibility criteria. These showed a large variety of factors associated with the use of hospital care services; models were found in only three studies. Age, gender, medical and nursing diagnoses, severity of illness, patient acuity, comorbidity, and complications were the characteristics found the most. Patient acuity and medical and nursing diagnoses were the most influencing characteristics. Models including medical or nursing diagnoses and patient acuity explain the variance in the use of hospital care services for at least 56.2%, and up to 78.7% when organizational factors were added.

**Conclusions:**

A larger variety of factors were found to be associated with the use of hospital care services. Models that explain the extent to which hospital care services are used should contain patient characteristics, including patient acuity, medical or nursing diagnoses, and organizational and staffing characteristics, e.g., hospital size, organization of care, and the size and skill mix of staff. This would enable healthcare managers at different levels to evaluate hospital care services and organize or reorganize patient care.

## Introduction

As health expenditures continue to rise, hospitals are challenged to provide more efficient and affordable services without compromising on quality. Efficient and high-quality hospital care is generally determined by three aspects. First, the size and educational level of the medical and nursing staff [1], [2]; second, the organization of care [3]; and third, the number of patients treated and their disease severity [4].

Because healthcare costs and consequently its affordability are related to the severity of a patient's condition (need for health care), and to the services requested (demand for health care), it is important for hospital managers to identify the factors that determine the demand [5]. If these factors could be identified, managers would be able to generate information on cost issues and substantiate trends in the demand for hospital care services over time. Furthermore, university hospitals could better define their top-referral patient populations and plan for capacity and capability through staff levels and facility planning.

At present, it is still unclear which individual, and preferably objective, patient characteristics are associated with the demand for hospital care services and their inherent costs. In recent attempts to reveal these characteristics, the focus was on specific patient populations [6], or different reference standards were used for analysing the characteristics and produced conflicting results [7].

When searching for associations between patient characteristics and the demand for hospital care services, it is necessary to define ‘demand for hospital care services’ or the product of this demand, i.e., ‘use of hospital care services’. Although the WHO defines ‘demand for health services’ as: *The health care expectations expressed by individuals or communities*, a more detailed interpretation of the term is lacking. For the purpose of this review, we further define the term ‘demand for hospital care services’ as the need for medical treatment and nursing care (i.e. personnel costs for medical and nursing staff as well as costs for therapeutic and diagnostic interventions), as determined by the individual patient's diagnosis and wishes.

During the nineteen-eighties and nineties, researchers put effort into matching the demand for hospital care services with nursing supplies. This was fuelled by economic pressures (i.e. nursing shortages [8] and the knowledge that the amount of nursing care needed varies substantially between diagnosis-related groups (DRGs) [9]). The above resulted in various definitions for ‘nursing care’ as well as various ways of predicting the demand for, or the measurement of nursing care actually given [10]. Clinical nursing care is most clearly expressed as ‘nurse hours per patient day’ (NHPPD) [11]. It is also customary to use the term for the product of the demand for care, i.e., ‘nursing care intensity’ or ‘workload’, [10], [12] as measured with a range of patient classification systems (PCS). In addition, other methods have been proposed, such as DRG nurse costing models or nurse-patient ratios [13]. Although these methods are commonly used, they have been criticized because nurses do not perceive them as a reflection of the ‘real’ nursing workload and these methods do not take into account changes in practice, e.g., a rise in care complexity or nursing care intensity [13], [14]. In addition, NHPPD, DRG costing models, and nurse-patient ratios are merely a proxy for the nursing care offered (personnel staffing) with the underlying assumption that all patients and all patient days are equal in terms of the use of health services.

In the medical world, the use of hospital care services is generally measured by costs for care as determined by DRG costing models [7], or length of stay (LOS) [15]. However, it is widely known that the intensity of patient care, and therefore the utilization of health services, increases as the LOS is shortened. Furthermore, LOS is substantially influenced by non-medical, for example, organizational factors [16], [17] and therefore not useful as an expression of the demand for medical services.

In the most favourable case scenario, the utilization of clinical hospital care services is defined as costs made during hospitalization, including the costs incurred for medical, nursing, diagnostic and therapeutic services. However, considering the variety of the measures and the shortcomings of some of them, we decided to study the use of hospital care services by using hospitalization costs, nursing workload and nursing care intensity. We therefore conducted a systematic literature review to search for associations between factors or models and the patient's demand for medical and nursing hospital care services in non high-care hospital wards.

## Methods

This systematic review was conducted according to the PRISMA Preferred Reporting Items for Systematic Reviews and Meta-analysis-statement [18].

### Eligibility criteria

Articles were eligible if they: 1) explored associations between health status parameters or patient characteristics and the demand for hospital care services; 2) focused on hospitalized patients on general wards; and 3) used regression or correlation analyses to explore possible associations.

We applied no restrictions on study design, but excluded other reviews including systematic reviews and original studies that merely described relative measures such as staffing levels, health outcomes, or length of hospital stay.

### Literature search and information sources

MEDLINE, Embase, CINAHL and Business Source Premier were searched from inception through June 2013 to find articles that predicted or explained the demand for hospital care services; there were no limits regarding publication status, date or language. The complete search strategy for each database is given in Appendix S1 (MEDLINE), Appendix S2 (Embase), and Appendix S3 (for CINAHL and Business Source Premier). The search was designed and conducted with the help of a clinical librarian.

### Study Selection

Eligible articles were independently selected by two reviewers (HV and DU) based on the relevance of their titles and abstracts as retrieved by the search. If articles met the inclusion criteria, full-text versions of the articles were obtained and further scrutinized for eligibility by CO and JHV. Authors were contacted for irretrievable articles. HV and DU also made the final selection of articles to be included. CO was involved in any cases of disagreement where consensus was reached through discussion. The reference lists of included articles were checked to detect any potential additional studies. Also, experts in healthcare services research were asked for potentially eligible studies.

### Study quality appraisal

The STROBE statement for cohort studies was used to assess the methodological quality of the included studies [19]. This standard contains general methodological aspects that are important and applicable to the studies included. Appraisal was undertaken by two reviewers independently (CO and JHV) and cross-checked afterwards. Quality items were judged as ‘−’ (not described) or ‘+’ (described) as according to the definition in the STROBE statement. Items scoring ‘+/−’ were partially present, e.g., when the study population was described in terms of the medical diagnosis rather than the patient characteristics.

### Data extraction and data items

Data extraction was performed by using a predefined, structured data-abstraction sheet and was double-checked during the process by CO and JHV. The following data were extracted: author, year of publication, setting, research design, sample size and specialty, (resource) reference standard, possible associated factors, measures of association with the demand for hospital care services, expressed as correlation coefficient (ρ), beta-coefficient (β) of a linear regression analysis, or odds ratio (OR) as derived from a logistic regression analysis, including their p-values and 95% confidence intervals (CI). We also documented whether the associations given had been corrected for other factors by means of a multivariable analysis. Where there was some uncertainty about the data, CO and JHV contacted the authors by e-mail.

### Data analysis

All models and factors in the included studies that were investigated for their association with the use of hospital care services were summarized. Associations were judged significant if P <0.05 or their CI did not enclose the value of 0 or 1.

Meta-analysis was intended if study designs, reference standards, and outcomes were homogeneous. Otherwise, the findings are described and categorized by the various models and factors found.

## Results

### Study selection and characteristics

**Figure 1:**
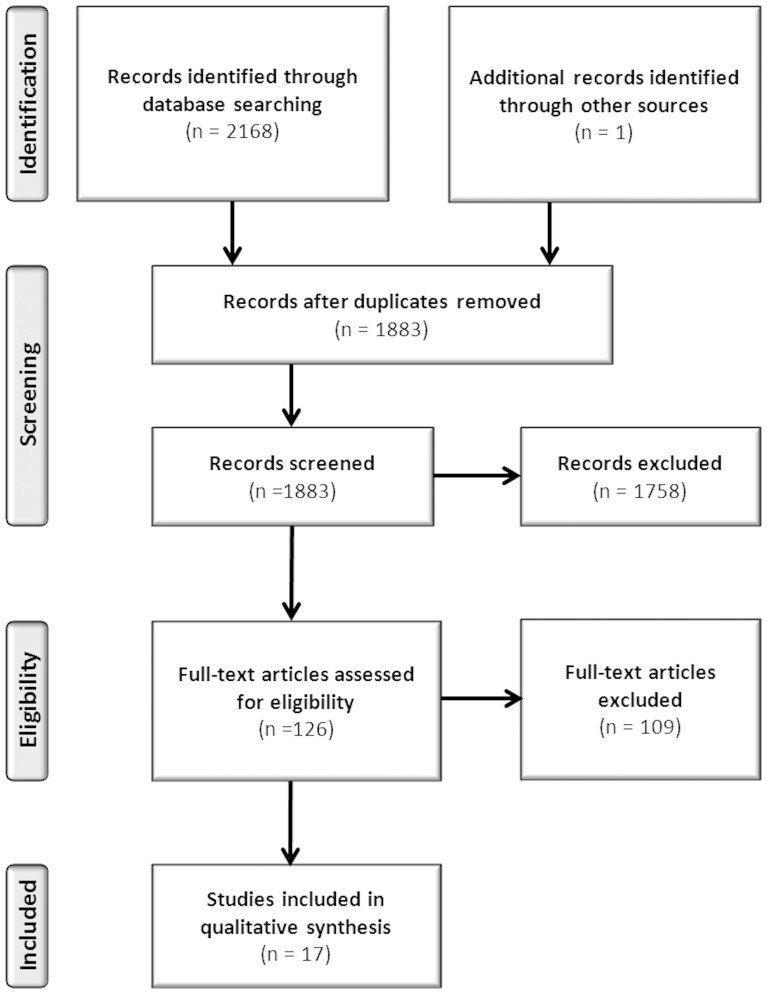
Summary of search strategy.

The search identified 2,168 studies from the four databases. After removing the duplicates and reviewing the titles and abstracts, 124 studies remained that met the inclusion criteria. Based on the full texts, a further 109 studies were excluded. Most of these studies (n = 83; 76%) did not report patient characteristics. For nine studies, all dissertations, the researchers received no reply to their queries for more information. Two authors replied to questions about their statistical analyses, but no extra data were obtained. One study was included after checking the references of one included publication. Another study was included because it was known by the researchers. Eventually, 17 studies were identified for this review (Figure 1).

The studies included (Table 1) were published between 1983 and 2013. Twelve out of the 17 studies (70%) had a retrospective design, while five studies (30%) were prospective cohort studies. Ten studies (59%) were conducted in the United States, five in Europe (30%) and two in Canada (11%). Data were taken from hospital sources including hospitalizations on different wards e.g. pulmonary, medical, surgery, obstetrics and gynaecology, intensive care, paediatrics, orthopaedics, geriatrics, and cardiology units. Study sizes ranged from 206 to 298,691 patients.

**Table 1:** Study characteristics.

Author	Setting	Design	N, specialty	Resource	Reference standard	Predictive factors	Results	Corrected	Statistical analysis
**Bostrom, 1991**	University hospital, United States, 600 beds	Retrospective	n = 1,372 patients	Medicus	average daily and total nursing hours per hospital stay	SII per DRG (14, 15, 89, 96, 138, 148, 182, 294, 320, 468)	daily range: r0.27 to r0.53	NA	correlation
							total range: r0.64 to r0.80		
**Bostrom, 1994**	University hospital, United States, 600 beds	Retrospective	n = 1,164 patients	Medicus	average daily and total nursing hours per hospital stay	SII per DRG (14, 15, 89, 96, 138, 148, 182, 294, 320, 468)	daily range: r^2^0.04; NS to r^2^0.30; p<0.001	corrected for physician practice	multivariable regression analysis
							total range: r^2^0.17; p<0.05 to r^2^0.49; p<0.001		
**Campbell, 1997**	Hospital, United Kingdom	Retrospective	n = 798 patients, respiratory medicine unit	TEAMWORK	weekly worked nursing hours	CMG cystic fibrosis	18%	NA	univariable regression analysis
									
**Caterinicchio, 1983**	8 hospitals (5 teaching), New Jersey, range 155–550 beds	Prospective	n = 2,660, medical-surgical, obstetric-gynaecologic, psychiatric and intensive care units	RNEUSI (grand total minutes corrected for skill level)	nursing resource use	Age	r0.2326; P>0.0001	NA	Pearson correlation analysis
									
**Fagerström, 2000**	Hospital, Finland	Prospective	n = 19,324 OPC records, on 8 units: 3 internal, 2 surgical, 1 gynaecological and 2 paediatric units	PAONCIL	daily nursing workload measure for ward organization	OPC	r^2^0.37		multivariable regression analysis
						Age per ward	r^2^0.001; NS	corrected for OPC score	
							r^2^0.09; p0.0488		
							r^2^0.064; p0.0008		
						Gender per ward	r^2^0.006;p0.37		
							r^2^0.000%; NS		
**Geissler, 2012**	712 hospitals, across 10 European countries: Finland, France, Germany, Spain, Sweden, Austria, Ireland, Poland, England and Estonia	Retrospective	n = 125,698 with hip replacement	national routine patient-level data samples from 2008	hospitalization costs, admission to discharge	Age 1 (1–60)	range r^2^0.068 to r2-0.004	corrected for DRG 1–14 (ordered by weight), DRG other, no. of procedures and adverse events	multivariable regression analysis
						Age 2 (61–70)	RC		
						Age 3 (71–75)	range β0.017 to β-0.082		
						Age 4 (76–80)	range β0.051 to β-0.049		
						Age 5 (>80)	range β0.070 to β0.051		
						Gender	range β0.026 to β-0.007		
						No. of diagnoses	range β0.036 to β-0.013		
						Transfer in	range β0.114 to β-0.125		
						Transfer out	range β0.112 to β-0.071		
						Emergency	range β0.117 to β-0.053		
						Deceased	range β0.346 to β-0.233		
						CCI 1	range β0.004 to β-0.030		
						CCI 2	range β0.137 to β-0.060		
						Urinary tract infection	range β0.178 to β-0.396		
						Wound infection	range β1.474 to β-0.027		
						Fracture	range β0.110 to β-0.06		
						Partial replacement	range β0.019 to β-0.318		
						Revisions of implants	range β0.399 to β0.154		
**Halloran, 1985**	Hospital, United States, 279 beds	Retrospective	n = 2,560 patients, every adult patient both admitted and discharged to one hospital	Rush-Medicus patient classification	daily nursing workload measure	Age	r0.198; p<0.001; <4%		correlation, stepwise multivariable regression analysis
						Marital status	NS		
						Payer	NS		
						Age, sex & race	r^2^0.043; p<0.001		
						DRG (3, 4, 11, 59, 75, 110, 121, 124, 132, 144, 156, 158, 189, 226, 227, 228, 264, 265, 266, 267, 271, 278, 282, 304, 322, 323, 348, 350, 355, 362, 382)	r^2^0.263; p<0.0001; range β17.855 to β-19.138		
						Nursing diagnoses & DRG	r^2^0.603; p<0.0001		
						Nursing diagnoses (37)	r^2^ 0.532; range β0.158 to β-0.093		
**Mahmoud, 2009**	196 hospitals, United States	Retrospective	n = 25,825 patients, adults undergoing elective colon procedures	Premier Perspective database	mean daily hospital costs (>US$15,000) (medical/surgical room and board, pharmacy, nursing, intensive care unit, central supply, laboratory, diagnostic imaging and operating room charges)	Surgical Site Infection	OR7.46 (CI 6.47–8.60)	corrected for antibiotic regimen	logistic regression analysis
						Age > 65years	OR 1.71 (CI 1.61–1.82)		
						Female sex	OR-0.87 (CI 0.8–0.93)		
						Caucasian race	OR-0.81 (CI 0.75–0.86)		
						BMI >30	OR 1.29 (CI 1.19–1.40)		
						High SENIC (risk of infection) score	OR 3.30 (CI3.02–3.70)		
**McMahon, 1992**	University hospital, Michigan, United States	Retrospective	n = 1,920 patients, ICU, general medicine and medical subspecialty units	RVU (costs without non direct patient costs)	hospital resource consumption	DRG (89, 96, 125, 127, 138, 140, 182, 183, 296, 410, 112, 124, 320)	r^2^0.10; p<0.0001		stepwise multivariable regression analysis
						DRG and FIRST (first APACHE-L in 24hours of admission)	r^2^0.14; p<0.0001; range r^2^0.18–r20.00		
						DRG and FIRST WORST (worst APACHE-L in 24 hours)	r^2^0.18; p<0.0001; range r^2^0.23–r20.00		
						DRG and WORST (value having highest APACHE-L weight during admission)	r^2^0.25; p<0.0001; range r^2^0.38–r20.00		
**Mion, 1988**	Cleveland Metropolitan General/Highland View Hospital, Canada	Prospective	n = 351 patients, 4 general medical units, 28 beds	PAS	total nursing workload score	PSI	r0.60; p<0.0001	corrected for LOS	Pearson's correlation, stepwise multivariable regression analysis
							r^2^0.48; p<0.0001		
						Age	r0.25; p0.0001		
						Gender	p<0.30		
						Race	p<0.30		
						Marital status	p<030		
						Admission source	r0.35, p0.0001		
						Discharge disposition	r0.54, p0.0001		
**O**'**Brien-Pallas, 1989**	University hospital, Canada, 1,000 beds	Prospective	n = 206 patients, diagnoses for diseases and disorders of the nervous system and circulatory system	GRASP; Medicus; PRN	daily and average nursing hours	CMG, LOS, age and sex			multivariable regression analysis
						PRN	r^2^0.58; p<0.0001		
						Medicus	r^2^0.56; p<0.0001		
						GRASP	r^2^0.57; p<0.0001		
**van Oostveen, 2013**	Academic medical center, The Netherlands, 1,000 beds	Prospective	n = 174 patients, surgical wards	time and motion research, hospital database	hospitalization costs (costs for physician services, nurse services, paramedics, all diagnostic tests, therapeutics, surgical procedures)	Age	β0.004; CI 0.001–0.007; p0.004		univariable regression analysis, stepwise multivariable regression analysis
						Gender (males)	β-0.015; CI −0.118–0.87; p0.767		
						Number of co-morbidities	β0.000; CI −0.031–0.030; p0.978		
						Number of complications	β0.221; CI 0.144–0.299; p0.000		
						**ASA-class**			
						1	RC		
						2	β0.168; CI 0.057–0.279; p0.003		
							RC		
						3	β0.234; CI 0.081–0.387; p0.003		
							β0.067; −0.071–0.204; p0.339		
						BMI at admission	β-0.006; CI −0.015–0.003; p0.189		
						Nutritional status	β0.018; CI 0.010–0.026; p0.000		
						Number of medications during hospitalization	β0.031; CI 0.022–0.040; p0.000		
						Admission type	β-0.210; CI −0.360–0.061; p0.006		
						**Surgical specialty**			
						TRAUMA	RC		
						URO	β0.776; CI 0.511–1.042; p0.000		
						ORTHO	β0.758; CI 0.505–1.012; p0.000		
						ABDO	β1.152; CI 0.900–1.405; p0.000		
						SHORT	β0.644; CI 0.368–0.920; p0.000		
						PLAST	β0.622; CI 0.381–0.943; p0.000		
						VASC	β0.786; CI 0.502–1.071; p0.000		
						ORAL	β0.679; CI 0.380–0.977; p0.000		
						Age, number of comorbidities, number of complications, number of medication during hospitalization, surgical specialty	r^2^0.562; p<0.000 - β0.002; CI 0.000–0.005; p0.072/β-0.038; CI −0.064–0.012; p0.005/β0.072; CI 0.005–0.139; p0.036/β0.013; CI 0.004–0.023; p0.007/ range β1.005 to β0.610; p<0.001		
**Sermeus, 2008**	115 acute hospitals, Belgium	Retrospective	n = 298,691 patients, ICU, surgical, internal medicine, geriatric and mixed surgical and internal medicine wards units	B-NMDS hospital financing and nurse staffing decisions	Prinqual 1; nurse care intensity	SJ	r^2^0.70		multivariable regression analysis
						Hospital type, hospital size, age, department type, DRG, severity of illness, DRG*severity of illness	r^2^0.40		
						SJ, hospital type, hospital size, age, department type, DRG, severity of illness, DRG*severity of illness	r^2^0.78		
**Shukla, 1992**	84 community hospitals, United States, average 196 beds ranging between 50 and 670 beds	Retrospective	n = 84 community hospitals, medical-surgical units	actual staffing and skill mix data using standard hourly wages	nursing costs by staffing/skill mix per ward per day	Patient acuity (GRASP)	r0.18; p0.19	NA	correlation
						CMI	r0.38; p<0.01		
				GRASP	Patient acuity	Age	r0.26; p<0.05		
						CMG	r0.12; p0.37		
**Titler, 2007**	One academic medical center, United States	Retrospective	n = 523 patients, >60 years older adults (568 hospitalizations) admitted for treatment for hip fracture or elective hip procedure	medical record database multiplied by cost to charge ratio hospital costs corrected for the fiscal year	hospital costs (general services, ICU/special care, pharmacy, laboratory, radiology, operating room, supplies and ancillary services)	Total number of medications	β0.0197; p<0.0001 (US$287,32 more costs)	corrected for nursing unit characteristics, medical treatments, individual treatments, individual medications, individual nursing interventions (fluid management, bathing, tube care and surgical preparation)	correlation, multivariable regression analysis - *only significant results given with direction of result of the correlation analysis
						Depression	Β-0.0943; p0.0078 (US$1299,59 lower costs)		
						Patient characteristics*			
						Gender	p0.2306		
						Age	p0.0003+		
						Religion	p0.7334		
						Race	p0.4908		
						Marital Status	p0.5109		
						Occupation	p0.0630		
						Severity of illness	p<0.0001+		
						Medical diagnoses*			
						Non traumatic joint disorders	p<0.0001−		
						Complications of device, implant or graft	p<0.0001+		
						Comorbidities*			
						Congestive heart failure	p0.0271+		
						Arrhythmias	p0.0137+		
						Valvular disease	p0.0043+		
						Pulmonary circulation disease	p0.0088+		
						Paralysis	p0.0098−		
						Other neurological disorders	p0.0077+		
						Diabetes	p0.0155+		
						Peptic ulcer disease without bleeding	p0.0404+		
						Lymphoma	p0.0409+		
						Metastatic cancer	p0.0189+		
						Coagulopathy	p0.0043+		
						Obesity	p0.0791−		
						Weight loss	p<0.0001+		
						Fluid and electrolyte disorders	p0.0102+		
						Chronic blood loss anaemia	p<0.0001+		
						Deficiency anaemia's	p0.1055+		
						Depression	p0.1263−		
**Titler, 2008**	Academic medical center in the Midwest, 843 beds	Retrospective	n = 1,075 patients, >60 years older heart failure patients (1,435 hospitalizations)	medical record database multiplied by cost to charge ratio hospital costs corrected for the fiscal year	hospital costs (costs for general services, ICU/special care, pharmacy, laboratory, radiology, operating room, supplies and other ancillary services)	Age	NS	corrected for nursing unit characteristics, multidisciplinary treatments, individual medications and nursing interventions	correlation, generalised estimate equations
						Gender	NS		
						Ethnicity	NS		
						Marital status	NS		
						Religion	NS		
						Occupation	NS		
						**Primary diagnosis**			
						Heart failure without hypertension	NS		
						Acute myocardial infarction	NS		
						Other cardiac conditions	NS		
						Conduction disorders	NS		
						Peripheral vascular disease	NS		
						Non-cardiac circulatory diseases	NS		
						Comorbidities			
						Deficiency anaemia	β0.0500; p0.483 (US$536.00 more costs)		
						**Severity of illness**			
						Severe	β-0.0318; p0.6355 (-US$327.22 lower costs)		
						Major	β-0.0062 ; p0.9187 (-US$64.62 lower costs)		
						Moderate	β-0.0840; p0.1699 (-US$842.29 lower costs)		
						Minor	RC		
						Total number of different medications	β0.017; p<0.0001 (US$179.24 more costs)		
**Wang, 2010**	US, dataset MarketScan Commercial Claims and Encounters inpatient	Retrospective	n = 23,216 heart failure related hospitalizations	dataset MarketScan Commercial Claims and Encounters inpatient	hospitalization costs (costs for physician services, all diagnostic tests, therapeutics, supplies and room fees)	Age		corrected for urban, region, LOS and secondary diagnosis	multivariable regression analysis
						18–39 years	US$388; p0.689		
						40–54 years	US$962; p0.038		
						55–64 years	RC		
						Gender	US$4316.7; p<0.001		
						CCI	US$229.5; p0.047		

B-NMDS  =  Belgium Nursing Minimal Data Set, BMI  =  Body Mass Index, CCI  =  Charlson Comorbidity Index, CMG = Case Mix Group, CMI  =  Case Mix Index, DRG  =  Diagnose Resource Group, GRASP  =  Grace Reynolds Application and Study of PETO, LOS  =  Length of Stay, NANDA =  North American Nursing Diagnosis Association, NA  =  not applicable, NS  =  not significant, OPC  =  Oulu Patient Classification, OR  =  Odds Ratio, PAS  =  Patient Acuity Scale, PAONCIL  =  Professional Assessment of Optimal Nursing Care Intensity Level, PRN  =  Project Resource Nursing, Prinqual 1  =  self-care (dependency level), PSI  =  Patient Severity Index, RC  =  reference category, RNEUSI  =  Registered nurse equivalents Units of Service index, RVU  =  relative value unit, SENIC  =  Study of the Efficacy of Nosocomial Infection Control, SII  =  Horn's Severity of Illness index, SJ = San Joaquin

From the 17 studies, various factors associated with the demand for hospital care services were investigated. These comprised patient characteristics [7], [12], [20], [21], [22], [23], [24], [25], [26], [27], Case Mix Group (CMG), DRG (Appendix S4), nursing diagnoses [7], [21], [24], [28], [29], [30] (Appendix S4), severity of illness [9], [22], [23], [25], [26], [30], [31], [32] (Appendix S5), patient acuity [12], [24], [30] (Appendix S5), comorbidities [7], [23], complications [7], [23], [25], [26], [33] and admission and discharge factors [22]. Three studies [21], [23], [30] investigated models estimating the demand for hospital care services.

Different outcomes were used to determine the amount of hospital care services demanded: five studies used nursing hours spent [9], [28], [29], [31], two studies used resource consumption [20], [32], three studies used nursing workload [12] or nursing workload as measured by a PCS [21], [22], Sermeus et al. [30] only used nursing care intensity, and seven studies used hospitalization costs [7], [23], [24], [25], [26], [27], [33]. Physician services, if investigated at all, were done so only indirectly.

As a result, only factors tested in multivariable analyses and individual factors (i.e. univariable and correlation analyses) are described. For the results of all univariable analyses and correlations between the utilization of hospital care services and associated factors please see Table 1. Because of large range of definitions of demand for health care services, we refrained from doing a meta-analysis.

### Methodological quality of studies

Overall, the methodological quality of the included studies was moderate to good (Table 2). Rationale, participants, variables and level of measurement, sample size and statistical methods were clearly reported. However, only eight (47%) studies mentioned their study design and provided an informative abstract. As most studies used large databases, the assessment of bias was hardly possible and limited to the data validation as reported by the investigators. Only six studies (35%) explained how missing data were handled, and in eight (56%) studies the characteristics of study participants were described. Seven studies that described the number of DRGs included, scored this as ‘partially present’ (31%). The precision of adjusted and unadjusted estimates was given in eight studies (47%).

**Table 2:** Methodological quality assessment.

STROBE items*	1	2	3	4	5	6^a^	7	8	9	10	12^a^	12^b^	12^c^	14^a^	16^a^	17	18	19	20	21	22
**Bostrom, 1991**	-	+	+	-	+	+	+	+	+/−	+	+	+	+	+/−	+/−	-	+	-	+	+	-
**Bostrom, 1994**	-	+	+	-	+	+	+	+	+	+	+	+	-	+/−	+	+	+	+	-	+	-
**Campbell, 1997**	+	+	+	+	+/−	+	+	+	+	+	+	+	+	-	-	-	+	+	+/−	+	-
**Caterinicchio, 1983**	+	+	+	+	+	+	+	+/−	+	+	+	+	+	+	+	+	+	-	-	+	+
**Fagerström, 2000**	+	+	+	+	+	+	+	+	+	+	+	+	+/−	NA	+/−	+	+	+	+/−	-	-
**Geissler, 2012**	+/−	+	+	+	+	+	+	+	-	+	+	+	-	+/−	+	+	-	+	-	-	-
**Halloran, 1985**	+	+	+	+	+	+	+	+	+	-	+	+	+	+	+/−	+	+	+	-	+	+
**Mahmoud, 2009**	+/−	+	+	+	+	+	+	+	-	+	+	+	-	+	+	+	+	+	+	-	+
**McMahon, 1992**	+/−	+	+	+	+	+	+/−	+	+	+	+	+	-	-	+/−	+	+	+	-	-	+
**Mion, 1988**	-	+	+	+	+	+	+	+	+	+	+	+	-	+	+/−	+	+	-	+	+	-
**O’Brien-Pallas, 1989**	+/−	+	+	+	+	+	+	+	+	+	+	+	-	-	+/−	+	+	+	+	+	-
**van Oostveen, 2013**	+	+	+	+	+	+	+	+	+	+	+	+	+	+	+	+	+	+	+	+	+
**Sermeus, 2008**	+	+	+	+	+	+	+	+	+	+	+	+	-	+	+/−	+	+	+	+	+	+
**Shukla, 1992**	+/−	+	+	+	+/−	+	+	+	-	-	+	+/−	-	+/−	-	+	+	+	+	+	-
**Titler, 2007**	+	+	+	+	+	+	+	+	+	+	+	+	+	+	+	+	+	+	+	-	+
**Titler, 2008**	+	+	+	+	+	+	+	+	+	+	+	+	-	+/−	+	+	+	+	+	+	+
**Wang, 2010**	+/−	+	+/−	+	+	+	+	+	+/−	+	+	+	-	+	+	+	+/−	+	+	-	+
**Percentage positive judgments**	**47%**	**100%**	**94%**	**88%**	**88%**	**100%**	**94%**	**94%**	**71%**	**88%**	**100%**	**94%**	**35%**	**56%**	**47%**	**88%**	**88%**	**82%**	**59%**	**65%**	**53%**

*1. title and abstract, 2. background, 3. objectives, 4. study design, 5. setting, 6. participants, 7. variables, 8. data sources/measurement, 9. bias, 10. study size, 11. quantitative variables, 12 statistical methods, 14^a^. descriptive data, 16^a^. main results, 17. other analyses, 18. key results, 19. limitations, 20. interpretation, 21. generalizability, 22. funding. Items 12^d^, 12^e^, 13, 14^ b^, 14^c^, 15, 16^b^ en 16^c^ were not applicable for assessing the included studies.

+  =  present, +/−  =  partially present, -  =  not present, NA.  =  not applicable.

### Models

Three models were found that could predict the use of hospital care services to a certain extent [21], [23], [30]. Halloran [21] reported a model comprising the patient's age, gender, and race, which explained only 4.3% of the nursing workload. In addition, Halloran described a model with nursing diagnoses and DRGs that explained 60% of the nursing workload as measured by a PCS. More than 20 years later, Sermeus et al. [30] could explain 78.7% of nursing care intensity as measured by a Nursing Minimal Data Set (NMDS) Prinqual 1, including hospital type, hospital size, department type, patient's age, San Joaquin system scores, DRG, and the interaction between DRG and severity of illness. By removing the San Joaquin scores, the model explained only 40.8% of nursing care intensity. Recently, van Oostveen et al. [23] reported a model comprising age, medication during hospitalization, complications, co-morbidity and medical specialty, explaining 56.2% of hospitalization costs for surgical patients.

### Individual patient characteristics

Five studies reported different results on the association between age and the use of hospital care services. Geissler et al. [7] reported a significant association between age and hospitalization costs (younger patients <61 years were more costly), while Mahmoud et al. [33] found older patients (>65 years) more likely to account for hospitalization costs over USD 15.000. Fagerström et al. [12] and Wang et al. [27] found that age contributed slightly but significantly to nursing workload and hospitalization costs. The study by Oostveen et al. [23] reported that age had no significant influence on hospitalization costs.

Three studies investigated the association of gender, race and BMI with costs. Geissler et al. [7] found lower costs for women than for men in three out of the seven countries investigated. This result was confirmed by Mahmoud et al. [33] and Wang et al. [27]. Additionally, Mahmoud et al. [33] found a decrease in costs for Caucasian patients and a cost increase for patients with a higher BMI score (>30).

### Diagnosis, DRG, CMG, case mix index & nursing diagnoses

DRGs and CMGs contributed 10% to hospital resource consumption [32] 18% to nursing hours [28], and 26.3% to nursing workload as measured by a PCS [21]. Sermeus et al. [30] performed a regression analysis including DRGs and a possible interaction between DRGs and severity of illness, but no significant interaction was found.

DRGs and nursing diagnoses together explained 60% of the variance for nursing workload as measured by a PCS. Nursing diagnoses alone contributed 53.2% [21]. One study [7] reported significantly more costs for hip replacement in patients with fractures (in three out of seven countries studied), lower costs in patients receiving a partial replacement (4/7 countries) and higher costs for revision of a hip implant (7/7) (Table 1). Van Oostveen et al. [23] found that the surgical specialties urology, orthopaedics, gastro-intestinal surgery, short-stay surgery, plastic surgery, vascular surgery and oral and maxillofacial surgery, as proxies for diagnosis, were more costly than trauma surgery. All specialties together explained 46% of the variance for hospitalization costs.

### Severity of illness/Physical health status

Severity of illness as measured by Susan Horns' Patient Severity Index (Appendix S5) contributed 48% to nursing workload as measured by a PCS [22]. The contribution of severity of illness to nursing hours varied widely per DRG (total range 17% to 49%) [31]. McMahon et al. [32] also found wide ranges for laboratory measurements, as a proxy for severity of illness, in the different DRGs. Although Titler et al. [26] showed a significant correlation between severity of illness and costs, they found no further significant differences in costs in their final model between different levels of severity.

### Patient acuity

Sermeus et al. [30] found the San Joaquin scores could explain most of the variance (70%) of nursing intensity, while Fagerström et al. [12] found their PCS contributed only 37% to nursing workload.

### Comorbidity and Complications

Two studies assessed comorbidity via the Charlson comorbidity index (CCI) in association with hospitalization costs [7], [27]. One of these studies found contradictory results [7] whereas Wang et al. [27] found an increase in hospitalization costs of USD 229.50 per index shift in the CCI. Patients with hip fractures and depression as comorbidity had reduced hospital costs by an average of USD 1299.59 [25]. In heart failure patients, only one comorbidity (deficiency anaemia) was associated with higher hospital costs (USD 536.00) [26]. The quantity of different medications being used by patients were also related to hospital costs [25], [26]. Geissler et al. [7] revealed higher costs for the total number of diagnoses as well as for urinary tract complications or wound infection. Van Oostveen et al. [23] reported significant effects of the total number of comorbidities −9%, complications +18%, and quantity of medications −3%, on hospitalization costs. For patients with high SENIC risk scores (Appendix S5) for surgical wound infections, the chance of costs rising above USD 15.000 was three times higher than in patients with low or moderate scores [33].

### Correlation

In five studies factors in their univariable or correlational analyses were used without testing them in multivariable analyses. Mion et al. [22] and van Oostveen et al. [23] reported a significant association between admission type (elective and emergency) and the hospital care services used. Mion et al. [22] also found a significant positive relationship for the type of discharge. Four research teams tested marital status [21], [22], [25], [26], religion and occupation [25], [26] as possible influencing factors, but no significance was found. The payer was also found not to influence nursing workload significantly [21].

The American Society of Anesthesiologists (ASA)-class was used by van Oostveen et al. [23] to measure the physical health status of patients. They found only two categories (1-2/1-3) of ASA-classes significantly associated with hospitalization costs.

Fourteen out of 30 specific comorbidities recorded in patients diagnosed with hip fractures were positively associated with hospital costs [25], while three comorbidities, i.e. depression, paralysis and obesity, showed a negative correlation. Primary diagnoses in heart failure patients were found not to influence hospital costs significantly [26].

## Discussion

This systematic review of 17 studies shows that the use of hospital care services is both defined and composed (i.e., financial components) differently across countries, disciplines and studies. Both organization-related and patient-related factors contribute to the use of hospital care services. In particular, age, gender, medical diagnosis, nursing diagnosis, severity of illness, patient acuity, comorbidity, and complications have been investigated the most and have been found to be associated significantly with the use of hospital care services.

The best combination of factors, explaining nearly 80% of the nursing care intensity, contained hospital type, hospital size, department type, age, severity of illness, DRG, and the San Joaquin system score [30]. However, this model contains patient characteristics as well as organizational factors, and explains nursing rather than medical services used. The second best model [23], containing only patient characteristics, explained 56.2% of the use of hospital care services. This implies that a combination of patient characteristics, including patient acuity, and organizational factors, results in the best model for explaining the use of hospital care services.

All models found examined individual patient characteristics as explanatory factors for the use of hospital care services, which suggests that these characteristics are important predictors for care demand. The characteristics found in this review can be used as predictors if they are known prior to a patient's admission, or as explanatory factors if they occur during admission, for example, to monitor trends in time regarding the demand for care. Therefore, the results of this review may be integrated into a practical dashboard for healthcare managers and policy-makers to manage and (re)organize their delivery of clinical hospital care at operational, tactic and strategic levels of decision-making. This will help substantiate their top-referral patient population, reorganize patient care, up-scale wards, planning budgets, capacity and capability, and evaluate the hospital care services themselves.

CMGs, DRGs and medical specialty [7], [23], [28], [30], [32] indicators for the medical diagnosis, were better suited for predicting the demand for hospital care services than the patient characteristics. Consequently, these indicators appear to be more suitable for explaining the use of hospital care services than individual diagnoses – apparently because the aggregate of this predictor corrects for variation at individual patient level. Nursing diagnoses [21] and the San Joaquin score for patient acuity [30], predicted the use of hospital care services even better than the indicators for the medical diagnosis. This seems plausible because nursing diagnoses and patient acuity scores contain similar elements regarding a patient's condition and aspects of nursing [21]. However, this characteristic cannot be derived easily from hospital databases, which poses difficulties to its practical application.

Contradictory results were found for factors like comorbidities and complications [7], [23], [25], [26], [27], [33]. In another review, Gijsen et al. stated that some negative associations found between comorbidity and the use of hospital care services may be due to the fact that the severity of the various comorbidities was not weighed in these studies [34]. Furthermore, less severe comorbidities may have been managed easily and less expensively with medication, while patients with more severe comorbidities may have had more expensive treatments.

One of the three models also addressed some organizational factors concerning hospital structure (e.g. hospital size, department type) [30]. Although the individual predictive values of most organizational factors were either not reported or small, they do determine efficient and high-quality hospital care [3]. Hence, these factors have to be included in any explanatory or prediction model for the use of hospital care services. This also holds for the size and educational level of the medical and nursing staff [1], [2], [35], but none of the studies in this review investigated these factors.

The limitations of this review are firstly, the heterogeneity of the reference standard ‘use of hospital care services’. Because hospitalization costs are defined differently in different countries, hospital databases are also set up differently resulting in the study aims being different. Hence, it is impossible to pool data and hardly possible to provide a clear result for each predictor. Secondly, the reference standard provides information on the amount of care delivered, which can be based on revenues rather than on the needs of patients [35]. Furthermore, the methodological quality of the included studies was fairly good, but 50% of the studies were somewhat dated. For instance, confidence intervals came into use during the nineteen-nineties [36] and were rarely reported earlier. Potential sources of bias and funding were also poorly reported, which may have flawed the validity of the results.

## Conclusion

This systematic literature review has revealed several patient characteristics that are significantly associated with the need or demand for healthcare services in the hospital setting. The most prominent characteristics were age, gender, medical diagnosis and nursing diagnosis, severity of illness, patient acuity, comorbidity, and complications, most of which can be derived from hospital databases. Complete models that explain the use of hospital care services should contain patient characteristics, including patient acuity, medical or nursing diagnoses, organizational factors and staffing characteristics, as these factors do determine efficient and high-quality hospital care, and therefore the costs of care. These models appear useful for healthcare managers and policy-makers as predictors or to monitor trends in time regarding the demand for care.

## Supporting Information

Appendix S1 **Search Embase.**
(DOC)

Appendix S2 **Search MEDLINE.**
(DOC)

Appendix S3 **Search CINAHL and Business Source Premier (EBSCO).**
(DOC)

Appendix S4 **DRG –explanation.**
(DOC)

Appendix S5 **Score explanation.**
(DOC)

Checklist S1 **PRISMA checklist.**
(DOC)
