# Supplementary material for: Factors and Models Associated with the amount of Hospital Care Services as Demanded by Hospitalized Patients: A Systematic Review
Source: PLoS One. 2014 May 30;9(5):e98102. doi: 10.1371/journal.pone.0098102 (PMC4039449; doi:10.1371/journal.pone.0098102)
Supplement: Appendix S1 — Search Embase. (DOC) [file pone.0098102.s001.doc]

**Appendix S1 Search MEDLINE**

Pà

(("Patients/classification"[Mesh] OR "Patients/statistics and numerical data"[Mesh] OR patient[tiab] OR inpatient*[tiab] OR "Inpatients/classification"[Mesh] OR "Inpatients/statistics and numerical data"[Mesh] OR "Inpatients/psychology"[Mesh])

AND

I🡪

("health status" [Mesh] OR "severity of illness index" [Mesh] OR “workload” [Mesh] OR workload measurement [tiab] OR care intensity [tiab] OR "Nursing Care/classification"[Mesh] OR "Nursing Care/organization and administration"[Mesh] OR "Nursing Care/statistics and numerical data"[Mesh] OR "Health Services Needs and Demand/classification"[Mesh] OR "Health Services Needs and Demand/statistics and numerical data"[Mesh] OR "Needs Assessment/classification"[Mesh] OR "Needs Assessment/organization and administration"[Mesh] OR "Needs Assessment/statistics and numerical data"[Mesh] OR patient dependency[tiab] OR patient acuity[tiab] OR patient characteristic*[tiab] OR health service use[tiab] OR patient clinical characteristic*[tiab]OR care requirement*[tiab] OR patient dependency level*[tiab])

AND

O🡪

("Classification/ methods"[Mesh] OR patient classification*[tiab] OR case mix[tiab] OR "Nursing Records/classification"[Mesh] OR "Nursing Records/statistics and numerical data"[Mesh] OR "Nursing Care/classification"[Mesh] OR "Nursing Care/organization and administration"[Mesh] OR "Nursing Care/statistics and numerical data"[Mesh] OR "Nursing Staff, Hospital/statistics and numerical data" OR "Health Services/statistics and numerical data"[Mesh] OR "Patient Care Planning"[Mesh] OR "Diagnosis-Related Groups/classification"[Mesh] OR "Diagnosis-Related Groups/statistics and numerical data"[Mesh] OR "Diagnosis-Related Groups/organization and administration"[Mesh] OR "Medical Records/classification"[Mesh] OR "Medical Records/statistics and numerical data"[Mesh] OR "Personnel Staffing and Scheduling"[Mesh] OR "Data collection"[Mesh] OR "Health Manpower"[Mesh] OR medical staffing[tiab] OR nurse staffing[tiab] OR physician staffing[tiab] OR requirement planning[tiab] OR workload measures[tiab] OR nursing hours per patient day[tiab] OR nursing workforce[tiab] OR physician workforce[tiab] OR nurse-patient ratio[tiab] OR patient-nurse ratio[tiab] OR nurse to patient ratio[tiab] OR patient to nurse ratio[tiab] OR physician to patient ratio[tiab] OR patient to physician ratio[tiab] OR physician-patient ratio[tiab] OR patient-physician ratio[tiab] OR "Medicare Assignment"[Mesh])

AND

(patient classification system [tiab]OR patient classification instrument* [tiab] OR "Nursing Assessment/methods"[Mesh] OR patient data management*[tiab] OR patient administration system [tiab] OR "Hospitalization/statistics and numerical data"[Majr] OR "Database Management Systems"[Mesh] OR "Hospital Information Systems"[Mesh] OR "Nursing Informatics/statistics and numerical data" [Mesh] OR "Medical Informatics/statistics and numerical data" [Mesh] OR "Forecasting" [Mesh] OR "Nursing Administration Research/statistics and numerical data" [Mesh]OR "Personnel Staffing and Scheduling Information Systems"[Mesh])

AND

("Validation Studies "[Publication Type] OR "Prospective Studies"[Mesh] OR Retrospective studies[Mesh] OR "Reproducibility of Results"[Mesh] OR "Task performance and analysis "[Mesh]OR "Regression Analysis"[Mesh] OR "Predictive Value of Tests"[Mesh] OR "Discriminant Analysis"[Mesh] OR "Costs and Cost Analysis"[Mesh] OR time management[tiab] OR work sampling[tiab]

NOT

("Treatment Outcome"[Mesh] OR "Outcome Assessment (Health Care)"[Mesh] OR "Hospital mortality"[Mesh])

NOT

("Critical care"[Mesh] OR "Intensive care"[Mesh] OR "Intensive care units" [Mesh] OR "Psychiatry"[Mesh] OR acute care[tiab] OR emergency department[tiab] OR recovery[tiab] OR psychiatry[tiab] OR dialysis[tiab]))
