# Supplementary material for: Factors and Models Associated with the amount of Hospital Care Services as Demanded by Hospitalized Patients: A Systematic Review
Source: PLoS One. 2014 May 30;9(5):e98102. doi: 10.1371/journal.pone.0098102 (PMC4039449; doi:10.1371/journal.pone.0098102)
Supplement: Appendix S2 — Search MEDLINE. (DOC) [file pone.0098102.s002.doc]

**Appendix S2 Search Embase**

.tw=text word

.ti.ab=title and abstract

.pt=publication type

P🡪

hospital patient/

aged hospital patient/

hospital$ adjusted patient$.tw

(hospitalized patient OR hospitalised patient).ti.ab

AND

I🡪

health status/

health status indicator/

disease severity/

workload/

workload.ti.ab

care intensity.tw

nursing care/

health care need/

(health care use OR healthcare use OR health service$ use OR health services needs and demand).tw

exp.needs assessment/

(patient dependency OR care requirement$ OR patient dependency level$).tw

patient acuity/

(patient characteristic* OR patient clinical characteristic*).ti.ab

AND

O🡪

nursing classification/

clinical classification/

patient coding/

casemix.ti.ab

diagnosis related group/

nursing care/

medical record/

nursing staff/

exp.hospital personnel/

health care manpower/

(nursing service$ OR physician service$ OR nursing service$, hospital).tw

(nurse staff$ OR medical staff$ OR requirement planning OR physician staff$ OR hospital staff$ OR personnel staffing and scheduling).tw

health care delivery/

workload measures.ti.ab

(nursing hours per patient day OR nursing workforce OR physician workforce OR nurse-patient ratio OR patient-nurse ratio OR nurse to patient ratio OR patient to nurse ratio OR physician to patient ratio OR patient to physician ratio OR physician-patient ratio OR patient-physician ratio).ti.ab

AND

(patient classification system OR patient classification instrument).ti.ab

nursing assessment/

(patient data management OR patient administration system$ OR

electronic medical record/

medical information system/

hospital information system/

nursing administration research/

exp.medical informatics/

exp.prediction and forecasting/

AND

health care cost/

hospitalization costs/

nursing costs/

exp.economic evaluation/

predictive value/

task performance/

exp.regression analysis/

discriminant analysis/

validation study/

prospective study/

retrospective study/

reproducibility/

NOT

exp.treatment outcome/

exp.mortality/

NOT

exp.intensive care/

exp.psychiatry/

psychiatric ward/

emergency ward/

(emergency care OR recovery OR dialysis).ti.ab
