# Supplementary material for: Factors and Models Associated with the amount of Hospital Care Services as Demanded by Hospitalized Patients: A Systematic Review
Source: PLoS One. 2014 May 30;9(5):e98102. doi: 10.1371/journal.pone.0098102 (PMC4039449; doi:10.1371/journal.pone.0098102)
Supplement: Appendix S3 — Search CINAHL and Business Source Premier (EBSCO). (DOC) [file pone.0098102.s003.doc]

**Appendix S3 Search CINAHL and Business Source Premier (EBSCO)**

P🡪

MH Inpatients /CL /SN

AND

I 🡪

MH Health status+

MH Workload

MH Workload Measurement

TX Care intensity

MH Nursing Care+ /AM /CL /EC /SN

MH Health Services Needs and Demand+ /CL /EC /SN

MH Needs Assessment +/CL

MH Nursing Assessment+ /AM /CL /SN

TI patient dependency OR AB patient dependency

MH [Patient Classification+ /AM /CL /SN](http://web.ebscohost.com/ehost/mesh/details?term=Patient Classification&sid=a22e58e9-ccf7-4f5d-b984-2e6aa48197a4@sessionmgr110&vid=71&hid=123)

TI patient acuity OR AB patient acuity

TI patient characteristic* OR AB patient characteristic*

TI care requirement* OR AB care requirement*

TI patient dependency level* OR AB patient dependency level*

AND

O🡪

MH Classification+ /MT

MH Patient Classification+ /AM /CL /SN

MH Patient Classification+ /MT /ST

TI casemix OR AB casemix

MH Medical Records+ /CL /SN

MH Nursing Care+ /AM /CL /SN

MH Medical Staff, Hospital+ /SN

MH Nursing Staff, Hospital+ /SN

MH Diagnosis-Related Groups+ /AM /CL /SN

MH Medical Records+ /CL /SN

MH Personnel Staffing and Scheduling+

MH Workload Measurement+ /CL/ EV /MT

TI medical staffing OR AB medical staffing

TI nurse staffing OR AB nurse staffing

TI physician staffing OR AB physician staffing

TI requirement planning OR AB requirement planning

TI nursing hours per patient day OR AB nursing hours per patient day

TI nursing workforce OR AB nursing workforce

TI physician workforce OR AB physician workforce

TI nurse to patient ratio OR AB nurse to patient ratio

TI patient to nurse ratio OR AB patient to nurse ratio

AND

MH Patient Classification+ /MT /ST

MH Workload Measurement+ /CL/ EV /MT

MH Nursing Assessment+ /MT

MH [Computerized Patient Record+](http://web.ebscohost.com/ehost/mesh/details?term=Computerized Patient Record&sid=a22e58e9-ccf7-4f5d-b984-2e6aa48197a4@sessionmgr110&vid=200&hid=1)

MH Hospitalization+ /SN

MH Management information systems+

MH Hospital information systems+

MH Health informatics+

MH Nursing administration research/ SN

TI patient data management OR AB patient data management

TI patient administration system OR AB patient administration system

AND

MH Instrument validation

MH Non experimental studies+

MH Task performance analysis+

MH Reproducibility of results

MH Retrospective design

MH Regression+

MH Predictive value of tests

MH Discriminant analysis

MH Cost and cost analysis

MH Cost control

NOT

MH Outcomes (Health care)+

MH Hospital mortality

NOT

MH Critical care+

MH Intensive care units+

MH Psychiatry+

TI acute care OR AB acute care

TI emergency department OR AB emergency department

TI recovery OR AB recovery

TI psychiatry OR AB psychiatry

TI dialysis OR AB dialysis
