# Supplementary material for: Factors and Models Associated with the amount of Hospital Care Services as Demanded by Hospitalized Patients: A Systematic Review
Source: PLoS One. 2014 May 30;9(5):e98102. doi: 10.1371/journal.pone.0098102 (PMC4039449; doi:10.1371/journal.pone.0098102)
Supplement: Appendix S4 — DRG –explanation. (DOC) [file pone.0098102.s004.doc]

Appendix S4 DRG-explanation

| **Author** | **DRG** | **description** | **n** | **Nursing diagnoses** |
| --- | --- | --- | --- | --- |
| **Bostrom, 1991** | 14 | Specific cerebrovascular disorders except TIA | 210 | NA |
|  | 15 | TIA & precerebral occlusions | 52 |  |
|  | 89 | Simple pneumonia & pleurisy age >17 with c.c. | 196 |  |
|  | 96 | Bronchitis & asthma. 17 with c.c. | 98 |  |
|  | 138 | Cardiac arrhythmia & conduction disorders with c.c. | 133 |  |
|  | 148 | Major small & large bowel procedures with c.c. | 207 |  |
|  | 182 | Esophagitis/ gastroenteritis & miscellaneous digestive disorders age >17 with c.c. | 148 |  |
|  | 294 | Diabetes age >35 | 58 |  |
|  | 320 | Kidney and urinary tract infections age >17 with c.c. | 79 |  |
|  | 468 | Extensive operating room procedure unrelated to principal diagnosis | 191 |  |
| **Bostrom, 1994** | 14 | Specific cerebrovascular disorders except TIA | NR | NA |
|  | 15 | TIA & precerebral occlusions | NR |  |
|  | 89 | Simple pneumonia & pleurisy age >17 with c.c. | NR |  |
|  | 96 | Bronchitis & asthma. 17 with c.c. | NR |  |
|  | 138 | Cardiac arrhythmia & conduction disorders with c.c. | NR |  |
|  | 148 | Major small & large bowel procedures with c.c. | NR |  |
|  | 182 | Esophagitis/ gastroenteritis & miscellaneous digestive disorders age >17 with c.c. | NR |  |
|  | 294 | Diabetes age >35 | NR |  |
|  | 320 | Kidney and urinary tract infections age >17 with c.c. | NR |  |
|  | 468 | Extensive operating room procedure unrelated to principal diagnosis | NR |  |
| **Halloran, 1985** | 3 | Infectious disease without secondary diagnosis | 22 | Altered level of consciousness |
|  | 4 | Infectious disease with secondary diagnosis | 23 | Less nutrition than required |
|  | 11 | Cancer of the GI system with surgery | 17 | Impairment of mobility |
|  | 59 | Benign tumour of the Uterus, Ovary with surgery | 22 | Decreased cardiac output |
|  | 75 | Diabetes without surgery with major secondary diagnosis | 23 | Altered self-concept: body image |
|  | 110 | Disease of the eye with surgical procedure | 20 | Depletion of body fluids |
|  | 121 | Disease of the heart- AMI | 32 | Thought process impaired |
|  | 124 | Disease of the heart, ischemia (except AMI) without surgery with major secondary diagnosis | 54 | Bowel constipation |
|  | 132 | Disease of the heart failure without surgery | 73 | Severe anxiety |
|  | 144 | Brain haemorrhage (Stroke) without surgery with major secondary diagnosis | 28 | Alteration of urinary pattern |
|  | 156 | Inflammation of the veins, blood clot without secondary diagnosis or with minor secondary diagnosis | 21 | Dysrhythmia of sleep-rest activity |
|  | 158 | Haemorrhoids | 20 | Actual Impairment of skin integrity |
|  | 189 | Upper GI disease except stomach ulcer without surgery with secondary diagnosis | 21 | Urinary incontinency |
|  | 226 | Disease of the gall bladder and the bile duct with surgery with age >50 | 28 | Bowel impaction |
|  | 227 | Disease of the gall bladder and the bile duct with surgery without secondary diagnosis | 17 | More nutrition than required |
|  | 228 | Disease of the gall bladder and the bile duct with surgery with secondary diagnosis with age <65 | 21 | Discomfort |
|  | 264 | Disease of the female reproductive system with surgical procedures without secondary diagnosis | 34 | Potential Impairment of skin integrity |
|  | 265 | Disease of the female reproductive system with surgical procedures with secondary diagnosis | 77 | Respiratory dysfunction |
|  | 266 | Disease of the female reproductive system with surgery | 65 | Excess body fluids |
|  | 267 | Benign breast tumour, chronic cystic disease without secondary diagnosis | 13 | Diarrhoea |
|  | 271 | Abortion without secondary diagnosis | 45 | Altered ability to perform hygiene |
|  | 278 | Delivery without surgery or with surgery assisting delivery | 228 | Noncompliance |
|  | 282 | Delivery with complications with Caesarean Section | 67 | Urinary retention |
|  | 304 | Backache, diffuse disease of connective tissue without surgery with secondary diagnosis | 22 | Pain |
|  | 322 | Indications of nervous, respiratory circulatory system disease without surgery without secondary diagnosis | 28 | Mild anxiety |
|  | 323 | Convulsions, fainting, nosebleed, chest pain without surgery with secondary diagnosis | 25 | Bowel incontinency |
|  | 348 | Fracture with major surgery | 18 | Altered composition of body fluids |
|  | 350 | Dislocation, sprains without surgery | 42 | Acute grieving |
|  | 355 | Internal injury of the skull, other organ without surgery with secondary diagnosis with age <41 | 20 | Delayed grieving |
|  | 362 | Open wound, multiple injuries without surgery with secondary diagnosis | 24 | Manipulation |
|  | 382 | Special admission with surgical procedure | 17 | Potential nutritional alternation |
|  |  |  |  | Confusion |
|  |  |  |  | Moderate anxiety |
|  |  |  |  | Panic |
|  |  |  |  | Anticipatory grieving |
|  |  |  |  | Sensory perceptual alternations |
|  |  |  |  | Altered ability to perform self-care |
| **McMahon, 1992** | 89 | Simple pneumonia and pleurisy, age >70 with complication and/ or comorbidity | 120 | NA |
|  | 96 | Bronchitis and asthma, age >70 with complications and/ or comorbidity | 53 |  |
|  | 112 | Vascular procedures except major reconstruction without pump | 710 |  |
|  | 124 | Circulatory disorders, excluding AMI with cardiac catheterization and complex diagnosis | 144 |  |
|  | 125 | Other circulatory disorders, cardiac catheterization | 59 |  |
|  | 127 | Health failure and shock | 148 |  |
|  | 138 | Cardiac arrhythmia and conduction disorders with c.c. | 68 |  |
|  | 140 | Angina Pectoris | 68 |  |
|  | 182 | Esophagitis/ gastroenteritis & miscellaneous digestive disorders age >17 with c.c. | 95 |  |
|  | 183 | Esophagitis/ gastroenteritis & miscellaneous digestive disorders age 18-69 | 23 |  |
|  | 296 | Nutritional and miscellaneous metabolic disorders age >17 with c.c. | 107 |  |
|  | 320 | Kidney and urinary tract infections age >69 and/ or c.c. | 88 |  |
|  | 410 | Chemotherapy | 256 |  |

AMI = Acute Myocardial Infarction, c.c.= comorbid conditions or complications, GI = Gastro Intestinal, NA = not applicable NR = not reported, TIA = transient ischemic attack
