# Supplementary material for: Factors and Models Associated with the amount of Hospital Care Services as Demanded by Hospitalized Patients: A Systematic Review
Source: PLoS One. 2014 May 30;9(5):e98102. doi: 10.1371/journal.pone.0098102 (PMC4039449; doi:10.1371/journal.pone.0098102)
Supplement: Appendix S5 — Score explanation. (DOC) [file pone.0098102.s005.doc]

Appendix S5 Score-explanation

| **Author** | **Score** | **Items** | **Response categories** |
| --- | --- | --- | --- |
| **Bostrom, 1991 & 1994** | SII | stage of principal diagnosis | 1 (least severe) to 4 (most severe) |
|  |  | complications of the principal condition |  |
|  |  | concurrent interacting conditions |  |
|  |  | dependency on hospital staff |  |
|  |  | extent of non-operating room procedure |  |
|  |  | rate of response to therapy or rate of recovery |  |
|  |  | remaining impairment after therapy |  |
| **Fagerström, 2000** | OPC | planning and coordination of care | 1 (least workload) to 4 (greatest workload) |
|  |  | breathing, blood circulation and symptoms of disease |  |
|  |  | nutrition and medication |  |
|  |  | personal hygiene and secretion |  |
|  |  | activity/ movement, sleep and rest |  |
|  |  | teaching/ guidance in care/ follow-up care and emotional support |  |
| **Mahmoud, 2009** | SENIC | operating room >2hours | 1 point/ 1 (low risk) |
|  |  | >3 discharge diagnoses | 1 point/ 2 (moderate risk) |
|  |  | abdominal surgery | 1 point/ 3 (high risk) |
| **McMahon, 1992** | APACHE-L | Haematocrit | Extracted from the original APACHEIII |
|  |  | Serum creatinine without ARF |  |
|  |  | Serum creatinine with ARF |  |
|  |  | Serum BUN |  |
|  |  | Serum Na+ |  |
|  |  | Serum albumin |  |
|  |  | Serum bilirubin |  |
|  |  | Serum glucose |  |
|  |  | pCO2 |  |
|  |  | pH |  |
| **Mion, 1988** | PAS | feeding | 1 (least workload) to 5 (greatest workload) |
|  |  | bathing, grooming and dressing |  |
|  |  | mobility |  |
|  |  | elimination |  |
|  |  | dressings and treatments |  |
|  |  | medication |  |
|  |  | mental status and behaviour |  |
|  |  | special needs |  |
|  | PSI | stage of the principal diagnosis | 1 (low severity) to 4 (high severity) |
|  |  | complications from the disease or from treatment |  |
|  |  | interactions of other illnesses |  |
|  |  | level of physical dependency |  |
|  |  | procedures |  |
|  |  | level of response to therapy |  |
|  |  | resolution of acute symptoms |  |
| **Sermeus, 2008** | SJ | Care relating to hygiene | No assistance, supportive assistance, partial assistance, complete assistance |
|  |  | Care relating to mobility | No assistance, supportive assistance, partial assistance, complete assistance |
|  |  | Care relating to elimination | No assistance, supportive assistance, partial assistance, complete assistance |
|  |  | Care relating to feeding | No assistance, supportive assistance, partial assistance, complete assistance |

APACHE-L = Acute Physiology and Chronic Health Evaluation-laboratory, OPC = Oulu Patient Classification, PAS = Patient Acuity Score, PSI = Patient Severity of Illness,

SENIC = Study of the Efficacy of Nosocomial Infection Control, SII = Horn’s severity of illness index, SJ = San Joaquin
